# High concordance of intraocular antibody synthesis against the rubella virus and Fuchs heterochromic uveitis syndrome in Slovenia

**Published:** 2012-12-01

**Authors:** Spela Stunf, Miroslav Petrovec, Nina Žigon, Marko Hawlina, Aleksandra Kraut, Jolanda D.F. de Groot-Mijnes, Nataša Vidovič Valentinčič

**Affiliations:** 1 Eye Hospital, University Clinical Center Ljubljana, Slovenia; 2 Institute of Microbiology and Immunology, Faculty of Medicine, University of Ljubljana, Sloveni; 3 University Medical Center Utrecht, The Netherlands

## Abstract

**Purpose:**

To prospectively study the relationship between Fuchs heterochromic uveitis syndrome (FHUS) and intraocular production of specific antibodies against the rubella virus (RV) in Slovenia.

**Methods:**

Using the Goldmann-Witmer coefficient technique, intraocular synthesis of specific antibodies against RV, herpes simplex virus, varicella-zoster virus, cytomegalovirus (CMV) and *Toxoplasma gondii*–specific immunoglobulin G antibodies was performed in 12 consecutive patients with clinically diagnosed FHUS and 12 patients with idiopathic recurrent unilateral anterior uveitis (AU) without clinical features of FHUS.

**Results:**

Specific intraocular antibody synthesis against RV with a positive Goldmann-Witmer coefficient was proven in 11 of 12 (92%) FHUS patients, and in none of the non-FHUS AU patients (Fisher’s exact test <0.0001). In one patient with FHUS, specific antibodies against RV and varicella-zoster virus were concurrently detected. Specific antibodies against cytomegalovirus were detected in one patient with unilateral recurrent AU.

**Conclusions:**

Intraocular production of specific immunoglobulin G against RV was proven in the majority of tested cohort of FHUS patients from Slovenia as compared to the group of patients with idiopathic AU, which suggests that RV is involved in the pathogenesis of FHUS in this geographic area.

## Introduction

Fuchs heterochromic uveitis syndrome (FHUS) is a chronic, low-grade, usually unilateral iridocyclitis that typically presents without acute signs of intraocular inflammation and in the absence of systemic disease. The etiology of FHUS was initially associated with different infections, specifically herpes simplex virus (HSV) [1], cytomegalovirus (CMV) [2], and *Toxoplasma* [3–5]. In the last decade however, it has come to be believed that FHUS is caused by rubella virus (RV) infection [6–11].

In clinical practice, the diagnosis of FHUS is complex, with an average delay of 3.7 years in 80% of patients; it may even take as long as 20 years to diagnose [12]. Only one-third of patients with FHUS fulfill the four main criteria: iris heterochromia or iris atrophy, stellate keratic precipitates, no posterior synechiae, and cataract; the other two thirds only exhibit 40 three of these symptoms [6,7]. De Visser et al. found that 53% of patients with RV-positive anterior uveitis (AU) lack iris heterochromia or iris atrophy [7]. Further, the various clinical signs of FHUS are not always present at the same time; moreover, in approximately 10% of cases, FHUS can be bilateral and misdiagnosed as intermediate uveitis [13]. When the clinical diagnosis is uncertain or needs further confirmation, exact microbiological diagnostic tools are essential. This is especially important so in cases of FHUS, because these patients do not need immunosuppressive therapy, topical steroids are not effective [14–16], and they have to be appropriately screened for glaucoma [12].

RV infection can present as isolated uveitis [11,16] and possibly FHUS [9,10]. It is well known that the introduction of vaccination against RV also resulted in a decline in FHUS [17]. However, only 10% of FHUS patients have a positive PCR for RV RNA [6].

In Slovenia, a strict program of compulsory vaccination against RV, including first dosage of the vaccine by the age of 12 to 18 months, was introduced in 1990. Between 1975 and 1990, only girls were uniformly vaccinated against RV at the age of 12 years. We report the results of a prospective study, in which we evaluated aqueous humor (AqH) analysis for RV in patients with clinically suspected FHUS.

## Methods

This was a two-year, cross-sectional, prospective association study, conducted in 2009 and 2010. Ethical approval for the research was obtained from the National Medical Ethics Committee of the Republic of Slovenia. Intraocular synthesis of RV-specific immunoglobulin G (IgG) was determined in 24 patients (24 eyes). Twelve patients had clinical findings consistent with FHUS, and were examined for the first time or as part of regular follow up at the uveitis clinic of the University Eye Hospital Ljubljana, Slovenia in 2009 and 2010. The other 12 patients had recurrent unilateral nonhypertensive idiopathic AU, and did not meet any of the clinical criteria for FHUS. They were examined at our clinic in the same period and served as the control group for the attempt to determine etiological reasons for the unilateral uveitis.

The classification of uveitis and grading of cells and flare were performed according to the criteria of the International Uveitis Study Group [18]. In the inclusion criteria for FHUS, it was necessary to have at least three of the four major typical clinical criteria for FHUS listed above (Table 1). All patients underwent screening for uveitis, including a detailed medical history, investigation of the erythrocyte sedimentation rate, complete blood count, measurement of serum angiotensin–converting enzyme levels, serologic tests for syphilis and *Borrelia*, chest radiography, Mantoux testing, and human leukocyte antigen B27 (HLA-B27) typing. Aqueous humor investigations were performed as part of our standard protocol, when an additional diagnostic tool is needed to disambiguate an uncertain diagnosis of chronic cyclitis. Anterior chamber paracentesis and AqH analysis were performed after patients’ signed informed consent had been given. Paired AqH and serum samples were obtained, and AqH sampling was performed using the standardized procedure described by van der Lelij [19]. Aqueous humor samples were taken with the help of a head magnifying lens while the patient lay supine on an operating chair. A lid speculum was used to spread the eyelids. Local anesthesia was given and the ocular surface was sterilized with povidone iodine and irrigated with 0.9% NaCl. The eye was than fixated firmly at limbus with Fluid Analysis Set (FAS) tweezers (L.KLEIN AG, Biel, Switzerland). A corneal pre- incision was made and up to 200 µl of AqH was aspirated with a 27 gauge tuberculin syringe.

**Table 1:** Clinical signs of uveitis in the Fuchs heterochromic uveitis syndrome group

Age at the time of AqH tap, gender	Vaccine against RV	Duration of uveitis before tap (years)	BCVA (Snellen)	Iris hetrochro-mia or atrophy*	KP*	Cata- ract*	IOL*	No syne- chia*	**	Glau-coma*	Vitritis*	GWC				
												RV	HSV	VZV	CMV	Toxo
55, F	no	13	1	0	1	0	1	1	3	1	1	44,65	<3/Neg	<3/Neg	<3/Neg	<3/Neg
43, F	12 yo	6	0,2	1	1	1	0	1	4	0	1	15,27	<3/Neg	<3/Neg	<3/Neg	<3/Neg
40, F	12 yo	3	1	1	1	0	0	1	3	0	1	38,06	<3/Neg	<3/Neg	<3/Neg	<3/Neg
50, M	no	7	0,3	1	1	0	1	1	4	1	1	38,43	<3/Neg	<3/Neg	<3/Neg	<3/Neg
33, M	no	3	0,5	1	1	1	0	1	4	0	1	7,78	<3/Neg	<3/Neg	<3/Neg	<3/Neg
71, M	no	3	0,8	1	1	0	1	1	4	0	1	<3/Neg	<3/Neg	<3/Neg	<3/Neg	<3/Neg
46, M	no	4	0,8	1	0	1	0	1	3	0	1	3,47	<3/Neg	<3/Neg	<3/Neg	<3/Neg
46, M	no	13	0,4	1	1	0	1	1	4	1	1	5,3	<3/Neg	<3/Neg	<3/Neg	<3/Neg
45, F	12 yo	16	0,6	1	1	1	0	1	4	1	1	47,74	<3/Neg	36,43	<3/Neg	<3/Neg
48, M	no	8	0,5	1	1	0	0	1	3	0	1	104,38	<3/Neg	<3/Neg	<3/Neg	<3/Neg
28, M	no	16	0,5	1	0	0	1	1	3	0	1	6,49	<3/Neg	<3/Neg	<3/Neg	<3/Neg
46, F	12 yo	5	0,6	1	1	1	0	1	4	0	1	5,55	<3/Neg	<3/Neg	<3/Neg	<3/Neg

*1-the sign present and 0-the sign absent, **number of positive signs for each patient (if the patient had cataract or was already operated a clinical sign of ‘cataract’ was considered positive), BCVA=best corrected visual acuity, M=male, F=female, CMV=cytomegalovirus, GWC=Goldmann Witmer coefficient, HSV=Herpes simplex viruses, IOL=intraocular lens, KP=keratic precipitates, neg=negative, pos=positive, RV=Rubella virus, Toxo=*Toxoplasmosis*, VZV=Varicela zoster virus, yo=years old.

Specific antibody titers against RV, HSV, varicella-zoster virus (VZV), CMV, and *Toxoplasma* in AqH and serum were determined at the Department of Virology, University Medical Center Utrecht, The Netherlands, using Enzygnost^®^ anti–rubella virus/IgG, anti-HSV/IgG, anti-VZV/IgG, anti-CMV/IgG, and anti-toxoplasmosis/IgG enzyme-linked immuno sorbent assay (ELISA) kits (Siemens, Marburg, Germany). The assays were performed essentially as described previously [9,20]. Briefly, serial two- and fourfold dilutions for aqueous humor and serum samples, respectively, were analyzed for specific IgG concentrations according to the manufacturer's instructions.

Total IgG titers in serum and AqH were determined through an in-house ELISA [14]. Intraocular antibody production was determined by calculating the Goldmann-Witmer coefficient (GWC; [specific IgG AqH/total IgG AqH]/[specific IgG serum/total IgG serum]) and was considered positive when the GWC value was more than 3. The independent *t* test was used for statistical comparison.

## Results

The patients’ age in the FHUS group was 28 to 71 years (mean 45.9±10.72), and in the control group it was 25 to 59 years (mean 43.16±10.69). The independent sample *t* test proved no statistical age difference between the groups (p=0.536, *t* test). There were seven male and five female patients in each group. None of the included FHUS patients were vaccinated in the vaccination program for babies; however, four females were vaccinated with the monovalent rubella vaccine at the age of 12 (Table 1).

In the FHUS group, the leading symptom was unilateral blurred or decreased vision with or without floaters and without pain. All patients had at least three of four typical clinical signs for FHUS; furthermore, all patients had vitritis and four patients had glaucoma. The clinical signs evident in the FHUS group are presented in Table 2.

**Table 2:** Clinical signs of uveitis in the idiopathic anterior uveitis group

Age at the time of aqueous tap, gender	Vaccination against RV	GWC				
		RV	HSV	VZV	CMV	Toxo
59, M	no	<3/Neg	<3/Neg	<3/Neg	<3/Neg	<3/Neg
48, M	no	<3/Neg	<3/Neg	<3/Neg	<3/Neg	<3/Neg
46, M	no	<3/Neg	<3/Neg	<3/Neg	15,64/Pos	<3/Neg
35, F	12 yo	<3/Neg	<3/Neg	<3/Neg	<3/Neg	<3/Neg
33, F	12 yo	<3/Neg	<3/Neg	<3/Neg	<3/Neg	<3/Neg
38, F	12 yo	<3/Neg	<3/Neg	<3/Neg	<3/Neg	<3/Neg
25, M	no	<3/Neg	<3/Neg	<3/Neg	<3/Neg	<3/Neg
54, M	no	<3/Neg	<3/Neg	<3/Neg	<3/Neg	<3/Neg
57, M	no	<3/Neg	<3/Neg	<3/Neg	<3/Neg	<3/Neg
44, M	no	<3/Neg	<3/Neg	<3/Neg	<3/Neg	<3/Neg
47, F	no	<3/Neg	<3/Neg	<3/Neg	<3/Neg	<3/Neg
32, F	12 yo	<3/Neg	<3/Neg	<3/Neg	<3/Neg	<3/Neg

M=male, F=female, CMV=cytomegalovirus, GWC=Goldmann Witmer coefficient, HSV=Herpes simplex viruses, neg=negative, pos=positive, RV=Rubella virus, Toxo=*Toxoplasmosis*, VZV=Varicela zoster virus, yo=years old.

All patients from the control group presented with idiopathic unilateral recurrent AU. The leading symptoms were mild to moderate pain, eye redness, and decreased visual acuity. The clinical picture included ciliary hyperemia, endothelial precipitates, cells 1+ to 2+, posterior synechiae, normal intraocular pressure, no cataract, no vitritis, and normal fundus exam.

Eleven of 12 FHUS patients (91.67%) had a positive GWC for RV. The GWC values were between 3.47 and 104.38, median 15.27 (Table 1). The FHUS patient with the negative GWC for RV was also negative for all other analyzed GWCs.

In the control group, none of the patients had a positive GWC for RV (p<0.0001, Fisher’s exact test). One patient, however, had a positive GWC for CMV with a GWC value of 15.64. One of the patients from the FHUS group had concurrent positive GWCs for RV and VZV.

## Discussion

A positive relationship between RV infection and the clinical picture of FHUS was confirmed in this prospective study for a cohort of Slovenian patients. The connection was suggested by published series from other regions [6–10].

We considered the clinical diagnosis of FHUS in patients with three or all four main clinical criteria for FHUS, and compared the results to a group of patients with clinically diagnosed unilateral recurrent idiopathic AU. The results of the study revealed high concordance of the clinical diagnosis of FHUS and a positive GWC (>3) for RV. The GWC for RV was positive in 11 of 12 FHUS suspects and in none of the controls.

There was a drop in FHUS observed in the United States after the introduction of the rubella vaccination program in that country [17], suggesting that vaccination can prevent the development of FHUS. On the other hand, the possibility that the live attenuated measles, mumps, rubella vaccines could cause FHUS cannot be disregarded entirely [21,22]. Universal vaccination against RV for children of both sexes at the age of 12 to 18 months has been mandatory in Slovenia since 1990. For some period before this date, only 12-year-old girls were vaccinated. None of the patients recruited in the study (FHUS or idiopathic anterior uveitis (IAU) group) was included in the vaccinated program for babies. A couple of females were vaccinated at the age of 12, so that vaccination is obviously not protective when it comes to developing FHUS. For all citizens of Slovenia born before 1989, RV should be considered as a causative microorganism in eye diseases such as FHUS.

One of the FHUS patients with a positive GWC for RV had a concurrent positive GWC for VZV. Concurrent production of specific antibodies against herpes viruses was reported in 11 of 63 tested FHUS patients by Ruokonen et al. [6]. One of their patients had a concurrent positive GWC for VZV, but he did not respond to systemic antiviral therapy. The authors proposed that in FHUS, RV represents the antigenic stimulus for an oligoclonal B-cell response with additional costimulation of other B cells. In this case, further specific antibodies can be found against other pathogens (such as HSV, VZV, and CMV) [6]. In our patient with concurrent positive GWC for RV and VZV, however, the situation was different. This patient also had high titers for HSV, but with a negative GWC. Therefore, we assume that the result showing concurrent intraocular production of antibodies specific to two different agents is a true result.

One patient with FHUS had a negative GWC for RV, but also for all other tested pathogens. He presented with the typical clinical picture of FHUS, specifically all four main clinical criteria of FHUS and vitritis. However, he was also 71 years old, which is beyond the typical age for FHUS. In older populations of Singaporese FHUS patients, in whom the GWC against RV was not tested, CMV was proposed as the causing agent [2]; the GWC for CMV was tested for this patient, and the result was negative. Possibly, there is another cause of FHUS, as was also proposed by de Visser [7]. FHUS is a name for a constellation of clinical symptoms, and may have different origins [23]. Thus, several other microorganisms have been proposed to be connected to FHUS [1,24,25].

Given the results discussed above, we would recommend the analysis of intraocular fluid, particularly AqH, in all cases of clinically suspected FHUS to confirm its association with RV and/or to search for alternative causes. The positive GWC for RV in our study implies the viral etiology of FHUS in each of the tested patients. In addition, the study consistently demonstrated the role of RV in FHUS, patients but not in patients with idiopathic recurrent unilateral AU (control group) in the only tertiary uveitis center in Slovenia, which is responsible for following up the majority of patients with a clinical presentation of FHUS in the country.
